# Conformité des formulaires de demande et des comptes rendus anatomopathologiques de pièces opératoires de cancer du sein au Bénin

**DOI:** 10.48327/mtsi.v3i4.2023.348

**Published:** 2023-12-05

**Authors:** Freddy Houéhanou Rodrigue GNANGNON, Falilatou SEIDOU, Christel Marie LALEYE, Fèmi Perez ODIDI, Arielle FLENON NAKOU, Josiane Angéline TONATO BAGNAN, Justin Lewis DENAKPO, Dismand Stephan HOUINATO, Dansou Gaspard GBESSI

**Affiliations:** 1 Clinique universitaire de chirurgie viscérale, Centre national hospitalier universitaire Hubert Koutoukou Maga (CNHUHKM), Cotonou, Bénin; 2 INSERM U1094, IRD U270, Université de Limoges, EpiMaCT (Épidémiologie des maladies chroniques en zone tropicale), Institut d’épidémiologie et de neurologie tropicale, OmegaHealth, Limoges, France; 3 Laboratoire d’épidémiologie des maladies chroniques et neurologiques (LEMACEN), Faculté des sciences de la santé de Cotonou, Université d'Abomey-Calavi (FSS-UAC), Bénin; 4 Laboratoire d'anatomie pathologique (LAPC), FSS-UAC, Bénin; 5 Laboratoire d'anatomie pathologique, Centre confessionnel Padre Pio, Cotonou, Bénin; 6 Centre hospitalier universitaire de la mère et de l'enfant Lagune, Cotonou, Bénin; 7 Clinique universitaire de gynécologie et d'obstétrique, CNHUHKM, Bénin

**Keywords:** Cancer du sein, Chirurgie oncologique, Conformité, Formulaire de demande d'examen, Compte rendu d'examen anatomopathologique, Bénin, Afrique subsaharienne, Breast cancer, Surgical oncology, Adequacy, Pathology request form, Histopathology report, Benin, Sub-Saharan Africa

## Abstract

**Introduction:**

Le cancer du sein requiert une prise en charge pluridisciplinaire. Les anatomopathologistes et les médecins communiquent grâce au formulaire de demande et au compte rendu d'examen anatomopathologique. Il est crucial que ces deux documents soient bien rédigés pour une bonne prise en charge des patientes.

**Objectif:**

Évaluer la complétude des formulaires de demande et des comptes rendus d'examen anatomopathologique de pièces opératoires de cancers du sein chez les femmes au Sud du Bénin.

**Méthode:**

Il s'agissait d'une étude transversale, descriptive et analytique, avec une collecte rétrospective de données sur 57 mois (4 ans et 9 mois). Nous avons utilisé les recommandations de la Haute Autorité de Santé de France comme référentiel et le logiciel SPSS pour traiter les données.

**Résultats:**

31,3% des formulaires de demande étaient conformes aux recommandations. Les comptes rendus étaient narratifs dans 92,7% des cas et 68,8% comportaient les critères minimaux. La présence d'emboles vasculaires, le statut HER2 et les récepteurs hormonaux étaient tous simultanément renseignés dans seulement 29,2% des comptes rendus. La présence d'emboles vasculaires était le facteur pronostique le plus souvent renseigné.

**Discussion:**

Les chirurgiens et les anatomopathologistes ne rédigent pas toujours entièrement les formulaires de demande et les comptes rendus d'examen anatomopathologique. Ceci peut s'expliquer par l'absence de référentiels nationaux, et les difficultés d'accès à l'immunohistochimie. L’élaboration de référentiels nationaux et l'utilisation de comptes rendus synoptiques pourraient améliorer les pratiques.

## Abstract (en)

**Introduction:**

Breast cancer requires multidisciplinary management. Pathologists and physicians communicate using the histopathology request form and the pathology report. There are some minimal criteria that both should respect.

**Objective:**

We assessed the adequacy of histopathology request forms and pathology reports in the management of female breast cancer specimens in Southern Benin.

**Method:**

This was a cross-sectional, descriptive and analytical study, with retrospective data collection over 57 months (4 years and 9 months). The adequacy of the histopathology request forms and pathology reports was assessed on the basis of the recommendations of the Haute Autorité de Santé (HAS) of France. Data processing was done using SPSS software. We checked frequencies with the Chi^2^ test, with a significance level set at 5%.

**Results:**

31.3% of histopathology request forms complied with HAS recommendations. Pathology reports were presented in a narrative way in 92.7% of cases and 68.8% met the minimal criteria. The presence of vascular embolus, of hormone receptors and the HER2 status were all reported in only 29.2% of the reports.

**Discussion:**

The draft of histopathology request forms and pathology reports did not comply to the required minimal criteria. This situation could mainly be explained by the inexistence of consensus between physicians and pathologists and by the lack of immunohistochemistry. Editing national referentials and using synoptic reports would give better results.

## Introduction

Avec plus de 2 millions de nouveaux cas et plus de 600 000 décès en 2020, le cancer du sein est un problème majeur de santé publique à l’échelle mondiale [26]. La situation semble encore plus préoccupante en Afrique subsaharienne. En effet, bien que l'incidence y soit parmi les plus faibles au monde, la mortalité y est la plus élevée.

Au Bénin, pays d'Afrique de l'Ouest, le cancer du sein est le premier cancer de la femme aussi bien en termes d'incidence que de mortalité [26]. Les raisons le plus souvent évoquées pour expliquer la surmortalité sont le diagnostic tardif et le faible accès aux traitements, mais également les dysfonctions du système de santé [15].

Le diagnostic de certitude et l’évaluation du pronostic du cancer du sein reposent sur l'examen anatomopathologique. Sa prise en charge se veut multidisciplinaire. La chirurgie est la pierre angulaire de ce traitement, particulièrement dans les pays en voie de développement où l'accès aux thérapies systémiques et à la radiothérapie peut se révéler problématique. En outre, l'information apportée par la pièce d'exérèse chirurgicale est capitale pour déterminer la prise en charge médicale et/ou radiothérapique adjuvante. Parallèlement, la complexité toujours croissante du traitement du cancer du sein, y compris le traitement chirurgical, exige un processus de diagnostic de haute qualité, dans lequel l'anatomie pathologique joue un rôle central.

Les chirurgiens et médecins oncologues d'une part, et les anatomopathologistes d'autre part, communiquent de manière formelle essentiellement au travers de deux documents: les formulaires de demande d'examen anatomopathologique (FDA) et les comptes rendus d'examen anatomopathologique (CRA). Les informations contenues dans le FDA relèvent du médecin préleveur (le chirurgien) et sont cruciales pour la bonne conduite de l'examen anatomopathologique. De même, les informations contenues dans les CRA de pièces opératoires sont d'une importance capitale pour le choix du traitement locorégional et systémique adjuvant et l'estimation du pronostic [5, 13, 14].

Il est donc crucial que ces documents soient bien rédigés pour permettre, d'une part aux anatomopathologistes d'effectuer un diagnostic précis des cancers et une évaluation optimale des facteurs pronostiques, d'autre part aux médecins oncologues de prendre les décisions adéquates. Les difficultés de communication sont les facteurs contribuant à la plupart des erreurs médicales entraînant un préjudice pour le patient; lorsqu'elles n'en sont pas la principale cause.

Dans les pays développés, les études sur le sujet sont nombreuses et la tendance est à la standardisation de ces documents [12, 25]. En France, la Haute Autorité de Santé (HAS) a édité des recommandations sur les données minimales à renseigner dans un CRA [29]. Cependant, il n'est pas toujours possible d'obtenir certaines de ces données dans les pays à faible revenu comme ceux d'Afrique subsaharienne. De plus, certaines de ces informations peuvent ne pas être pertinentes au vu des moyens thérapeutiques disponibles dans ces pays [31]. Par conséquent, des instances internationales telles que la Breast Cancer Initiative 2.5 (BCI 2.5) ont stratifié les recommandations en fonction des contextes et des ressources disponibles dans chaque pays (ressources de santé de niveau basique, moyen, amélioré et maximal) [2, 10, 20].

En Afrique subsaharienne, à notre connaissance, peu d’études ont évalué les pratiques des chirurgiens et des anatomopathologistes dans la rédaction de ces documents. De plus, au Bénin, il n'existe pas de recommandations nationales concernant la présentation des FDA et des CRA.

Notre objectif est d’évaluer la complétude des données renseignées dans les FDA et les CRA des pièces opératoires des patientes prises en charge pour un cancer du sein dans deux hôpitaux de référence au Bénin.

## Cadre et méthode d’étude

### Type d’étude, cadre et population

L’étude était transversale, descriptive et analytique, avec une collecte rétrospective des données. Elle a intéressé les FDA et les CRA de pièces opératoires de cancer du sein histologiquement confirmé, édités entre le 1^er^ janvier 2015 et le 30 septembre 2020. La structure des FDA différait en fonction des services; de même, la structure des CRA différait en fonction des laboratoires et du pathologiste qui les rédigeaient. Ces documents étaient établis par les équipes médicales de chacune de ces structures.

Nous avons effectué une étude multicentrique incluant deux hôpitaux de référence à l’échelle nationale dans la prise en charge des cancers du sein, situés à Cotonou (capitale économique du Bénin):

- Centre national hospitalier universitaire Hubert Koutoukou Maga (CNHU-HKM)
- Centre hospitalier universitaire de la mère et de l'enfant Lagune (CHU-MEL)

Les observations médicales de patientes ayant un cancer du sein histologiquement confirmé et ayant bénéficié d'une chirurgie mammaire, conservatrice ou radicale, et pour lesquelles un CRA était disponible, ont été recensées dans un premier temps. Les FDA correspondants étaient ensuite recherchés dans les laboratoires d'anatomie pathologique desservant ces hôpitaux de référence. Il s'agissait d'un laboratoire public – le laboratoire d'anatomie pathologique et de cytologie de la Faculté des sciences de la santé (LAPC-FSS) – et de trois laboratoires privés – le laboratoire d'anatomie pathologique et de cytologie du Centre Adéchina/Clinique Dubois (CAAP), le laboratoire d'anatomie pathologique du cabinet médical Foi en Dieu et le laboratoire d'anatomie pathologique du Centre confessionnel Padre Pio. Tous les laboratoires étaient situés dans la ville de Cotonou.

Les FDA et CRA de biopsie ont été exclus.

### Variables

Dans un premier temps, la complétude des formulaires de demande et des comptes rendus d'examen anatomopathologique a été évaluée, selon les recommandations de la HAS de France. Seul l'aspect quantitatif a été évalué.

Les critères de la HAS sont répartis en deux groupes [29]:

- **Description de la pièce opératoire**
  Elle est sous la responsabilité du médecin préleveur (le chirurgien). Cette description comprend quatre éléments qui ne peuvent être renseignés par le pathologiste et qui lui ont été transmis par le chirurgien. Nous avons donc recherché ces éléments, directement dans les FDA transmis par les chirurgiens aux différents laboratoires d'anatomie pathologique. Ces éléments sont:
  1. la latéralité du sein;
  2. la localisation de la tumeur dans le sein;
  3. la technique chirurgicale employée;
  4. l'orientation de la pièce opératoire.
- **Description histopathologique**
  Elle a directement été recherchée dans les CRA. Cinq éléments doivent être renseignés au minimum selon les recommandations de la HAS [29]:
  1. le type histologique [17];
  2. le grade histopronostique [9];
  3. l'extension tumorale (marge d'exérèse, existence de foyers infiltrants multiples) [29];
  4. les critères permettant de déterminer le pT/pN (statut des ganglions axillaires) [17];
  5. les autres facteurs pronostiques et /ou prédictifs:
    - emboles vasculaires [29];
    - statut des récepteurs hormonaux (œstrogènes et progestérone) par immunohistochimie [29];
    - statut HER2 par immunohistochimie [29];
    - réponse histologique après traitement néo-adjuvant [23].

Nous avons par ailleurs distingué deux types de mode de présentation des CRA:

- CRA narratifs
- CRA synoptiques

Étaient considérés comme synoptiques, les CRA contenant un tableau synthétique présentant les résultats de l'examen [4]. Tous les autres étaient considérés comme narratifs. Dans un second temps, les CRA ont été répartis en trois groupes selon la classification du Breast Cancer Initiative 2.5 qui classe les outils employés dans la prise en charge du cancer du sein en quatre niveaux [1, 2, 18, 24]. Ainsi avons-nous distingué, en ce qui concerne le diagnostic:

- les analyses de niveau basique regroupant des éléments qui devraient être renseignés dans tous les CRA quel que soit le niveau de ressources dont dispose le système sanitaire. Il s'agissait: du type histologique, du grade SBR et de la classification pT/pN [8, 10].
- les analyses de niveau moyen regroupant des éléments qui peuvent être renseignés en utilisant des ressources limitées mais qui induisent une amélioration majeure de la prise en charge. Il s'agit de l’évaluation des marges, de la recherche d'emboles lymphovasculaires et de la recherche des récepteurs aux œstrogènes [8, 10].
- les analyses de niveau amélioré regroupant des éléments qui nécessitent des ressources importantes et qui produisent une amélioration de la prise en charge. Il s'agit de la recherche des récepteurs à la progestérone et HER2 [8, 10].

La Breast Cancer Initiative 2.5 définit un quatrième niveau d'outils, les « analyses de niveau maximal », qui n'est pas pertinent pour cette étude réalisée dans un pays à ressources limitées où l'accès à certaines options thérapeutiques de dernière génération est impossible [21].

### Analyse statistique

La comparaison des fréquences a été réalisée avec le test de Chi^2^ lorsque les effectifs théoriques étaient supérieurs ou égaux à 5. Lorsque les conditions d'application n’étaient pas remplies, le test de Fischer a été utilisé. Le seuil de significativité des tests statistiques a été fixé à 5%. Le traitement des données a été réalisé à l'aide du logiciel SPSS version 26.0.

## Résultats

**Figure 1:**
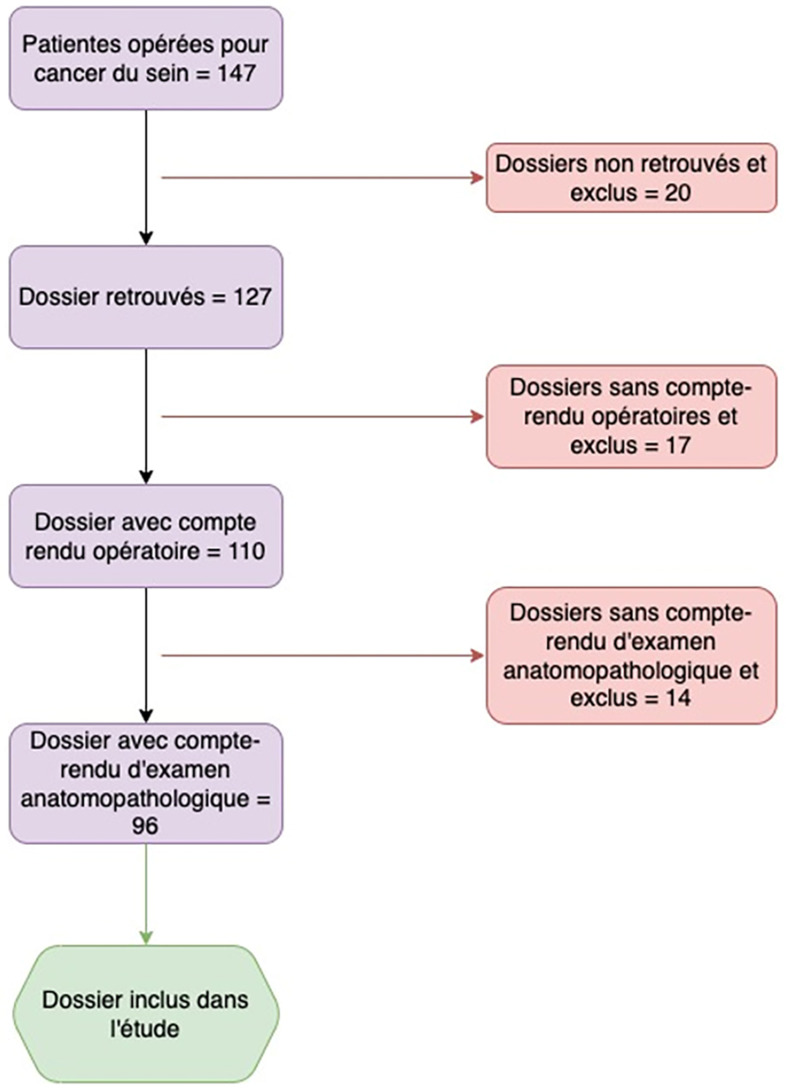
Organigramme de l’étude Flow-chart of the study

**Figure 2:**
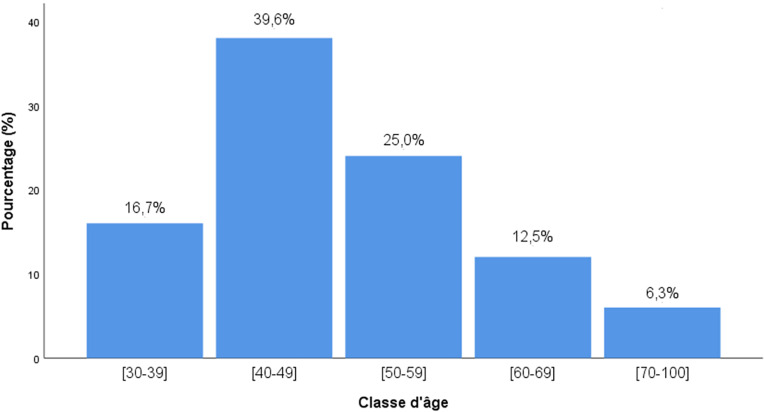
Répartition des patientes en fonction de l’âge au diagnostic (n = 96) Distribution of patients by age at diagnosis (n = 96)

Durant la période d’étude, 147 patientes ont bénéficié d'une chirurgie pour cancer du sein dans les deux centres de référence. Les observations médicales de 51 d'entre elles (35%) ne remplissaient pas les critères d'inclusion. Finalement, 96 observations médicales ont été considérées pour l'analyse finale (Fig. 1). L’âge moyen des patientes au moment du diagnostic était de 49,2 ans avec un écart-type de 11 ans. Le pic de fréquence était observé entre 40 et 50 ans (Fig. 2). L'intervention chirurgicale la plus réalisée était la mastectomie radicale associée à un curage axillaire (n = 61; 63%). La majorité des pièces opératoires étaient orientées (n = 68; 71%) avec des marges saines (n = 78; 81%) à l'analyse histopathologique. Le type histologique prédominant était le carcinome infiltrant de type non spécifique (n = 88; 92%). Les caractéristiques générales des 96 pièces opératoires sont détaillées dans le Tableau I.

**Tableau I:** Caractéristiques générales des pièces opératoires de cancer du sein General characteristics of breast cancer surgical specimen

Caractéristiques		Fréquence	%
**Sein atteint**			
	droit	52	54,1
	gauche	40	41,7
	bilatéral	0	0,00
	non documenté	4	4,2
**Quadrant du sein atteint**			
	central	1	1,0
	quadrant supéro-externe	11	11,5
	quadrant supéro-interne	1	1,0
	quadrant inféro-interne	2	2,1
	quadrant inféro-externe	6	6,3
	multiples	20	20,8
	non documenté	55	57,3
**Curage axillaire**			
	au moins 10 ganglions	61	63,5
	moins de 10 ganglions	17	17,8
	non réalisé	18	18,7
**Type histologique**			
	carcinome canalaire in situ	1	1,0
	carcinome infiltrant de type non spécifique	88	91,7
	carcinome lobulaire infiltrant	2	2,1
	carcinome mucineux	1	1,0
	réponse histologique complète	4	4,2
	non documenté	0	0,00
**Grade SBR**			
	I	13	13,5
	II	44	45,8
	III	33	34,4
	non documenté	6	6,3
**Récepteur aux œstrogènes**			
	négatif	20	20,8
	positif	21	21,9
	non documenté	55	57,3
**Récepteur à la progestérone**			
	négatif	27	28,1
	positif	14	14,6
	non documenté	55	57,3
**Statut HER2**			
	négatif	35	36,4
	positif	6	6,3
	non documenté	55	57,3
**Embole lymphovasculaire**			
	absent	53	55,2
	présent	14	14,6
	non documenté	29	30,2
**Taille de la tumeur T selon la classification pTNM**			
	pT0	3	3,1
	pT1	8	8,3
	pT2	31	32,3
	pT3	15	15,6
	pT4	6	6,3
	non documenté	33	34,4
**Ganglions N selon la classification pTNM**			
	pN0	25	26,1
	pN1	15	15,6
	pN2	12	12,5
	pN3	5	5,2
	pNx	1	1,0
	non documenté	38	39,6
**Marges**			
	saines	78	81,2
	atteintes	12	12,5
	non documenté	6	6,3
**Type de compte rendu**			
	narratif	89	92,7
	synoptique	7	7,3

### Formulaire de demande d'examen anatomopathologique

**Figure 3:**
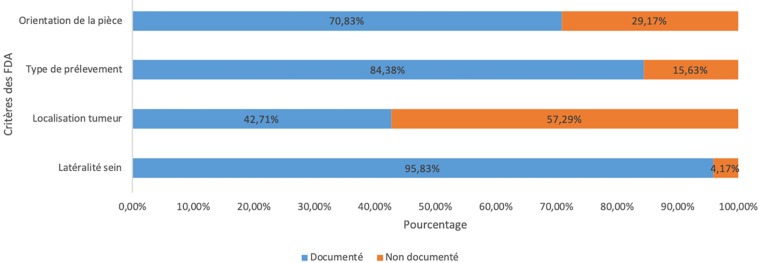
Fréquence d'apparition des différents critères minimaux sur les formulaires de demande d'examen anatomopathologique (n = 96) Frequency of minimal criteria on histopathology request forms (n = 96)

La situation de la tumeur dans le sein était l’élément le moins souvent renseigné (n = 41; 43%). L’élément le plus souvent renseigné était la latéralité du sein (n = 92; 96%). La Figure 3 résume la fréquence des principaux critères sur les FDA.

### Compte rendu d'examen anatomopathologique

#### Mode de présentation

Les CRA étaient narratifs dans 93% (n = 89) des cas. Nous avons retrouvé 7 comptes rendus synoptiques soit 7%.

#### Éléments renseignés

**Figure 4:**
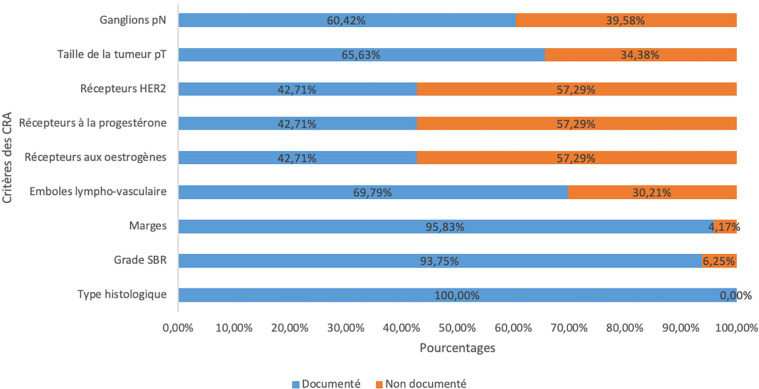
Fréquence d'apparition des différents critères minimaux sur les comptes rendus d'examen ana-tomopathologique (n = 96) Frequency of minimal criteria on pathology reports (n = 96)

Conformément aux critères d'inclusion, un type histologique était présent sur tous les comptes rendus sauf pour 4 patientes (4,2%) qui avaient présenté une réponse histologique complète sans doute suite à une chimiothérapie néo-adjuvante. La marge (n = 92; 96%) était le critère le plus souvent renseigné par les pathologistes sur les CRA, suivi du grade histopronostique selon la classification de Scarff Bloom Richardson (n = 90; 94%). Les critères explorés par l'immunohistochimie (récepteurs aux œstrogènes, récepteurs à la progestérone, surexpression du HER2) étaient les critères les moins souvent renseignés (Fig. 4).

En ce qui concerne la présentation, les CRA synoptiques renseignaient plus souvent les critères immunohistochimiques (récepteurs à l’œstrogène, récepteurs à la progestérone, surexpression du HER2) que les CRA narratifs. Cette différence était statistiquement significative (p < 0,05). Le Tableau II résume la répartition des éléments renseignés (narratif/synoptique) en fonction du type de CRA.

**Tableau II:** Répartition des fréquences de renseignement des critères minimaux selon le type de compte rendu Distribution of the frequencies of minimal criteria according to report type

		**Type de compte rendu**				
		**Narratif (n=89)**		**Synoptique (n=7)**		**p**
		**Fréquence**	**%**	**Fréquence**	**%**	
Analyses de niveau basique						
	taille de la tumeur T	60	67,4	7	100	0,071
	ganglions N	63	70,8	7	100	0,094
	type histologique	83	100	7	100	
	grade SBR	83	93,3	7	100	0,478
	1 élément présent	2	2,2	0	0	NA
	2 éléments présents	24	27	0	0	NA
	3 éléments présents	7	7,9	0	0	NA
	4 éléments présents	56	62,9	7	100	NA
Analyses de niveau moyen						
	récepteurs aux œstrogènes	34	38,2	7	100	0,001
	emboles lymphovasculaires	61	68,2	6	85,7	0,402
	marges	85	95,5	7	100	0,567
	0 élément présent	3	3,4	0	0	NA
	1 élément présent	15	16,9	0	0	NA
	2 éléments présents	48	53,9	1	14,3	NA
	3 éléments présents	23	25,8	6	85,7	NA
Anayses de niveau amélioré						
	statut HER2	34	38,2	7	100	0,001
	récepteurs à la progestérone	34	39,5	7	100	0,001

NA: Non applicable

## Discussion

### Intérêt et limites de l’étude

Cette étude sur la complétude des FDA et des CRA de pièces opératoires de cancer du sein dans deux hôpitaux de référence du Bénin a mis en évidence une discordance entre les pratiques et les recommandations pour un pays à faibles ressources comme le Bénin.

Toutefois, notre étude présente des limites. D'une part, réalisée dans deux hôpitaux publics de référence à l’échelle nationale situés dans le Sud du Bénin, elle n'est pas représentative de la situation dans tous les hôpitaux du pays, notamment les centres publics du Nord du pays et les centres privés. D'autre part, les insuffisances dans le processus d'archivage nous ont contraints à exclure les observations de certaines patientes. Ceci a considérablement réduit la taille de notre échantillon, diminuant la puissance statistique de notre étude.

Malgré ces limites, notre étude revêt un intérêt certain. En effet, dans les pays développés, de nombreuses études ont évalué l'exhaustivité des renseignements contenus dans les CRA de pièces opératoires de cancer du sein et ont par conséquent permis de faire des recommandations qui ont abouti à une meilleure rédaction de ces documents [4, 6, 12, 16, 19, 22, 25, 30].

En Afrique subsaharienne, par contre, peu d’études ont été réalisées. À notre connaissance, seules trois études publiées ont décrit des audits similaires. Ces trois études ont identifié des lacunes importantes dans le renseignement des informations provenant des analyses du niveau basique du BCI 2.5 sur les FDA et les CRA [3, 7, 31]. Autant que nous le sachions, notre étude est la première au Bénin à évaluer les FDA et CRA dans les cancers du sein. Elle pourra servir de référence pour la réalisation d’études plus importantes.

### Formulaire de demande d'examen anatomopathologique

Nous avons déterminé que seulement 31% des FDA contenaient l'ensemble des quatre renseignements minimaux requis par la HAS. L'absence de ces renseignements peut influer sur le délai et la qualité de l'examen anatomopathologique, notamment en ce qui concerne l'orientation de la pièce opératoire et la localisation de la tumeur dans le sein. En effet, en l'absence de ces renseignements, le pathologiste doit recontacter le médecin préleveur avant de procéder à l'examen. Dans notre contexte où les pathologistes sont surchargés, car peu nombreux [11], ces situations contribuent à prolonger le délai de rendu des résultats. Nos résultats sont similaires à ceux obtenus par Vallacha *et al.* à Karachi (Pakistan), qui ont trouvé 34% de FDA contenant les quatre critères minimaux [28]. En revanche, Barré *et al*., dans une étude prospective, ont retrouvé au CHU de Nantes que 69% des FDA étaient complets. L’élément le moins souvent renseigné dans cette étude était la technique chirurgicale utilisée [6]. Les différences entre nos résultats et ceux de Barré *et al.* pourraient s'expliquer par les différences de méthodologie, et surtout par le fait que le FDA utilisé dans le CHU de Nantes était un formulaire prérempli, plus facile à renseigner.

### Compte rendu d'examen anatomopathologique

L'absence de certains renseignements dans un CRA de pièce opératoire peut induire une décision inadéquate pour le choix du traitement adjuvant. Ainsi, l'absence d'information sur les récepteurs hormonaux sur le CRA prive d'emblée les patientes de l'accès à l'hormonothérapie dans le cas, plutôt fréquent, où la recherche des récepteurs hormonaux n'a pas été effectuée sur la pièce de biopsie initiale. Nous avons remarqué une grande variabilité dans la fréquence des différents critères présentés dans les comptes rendus d'examen anatomopathologique. Globalement, les éléments fournis par une analyse histologique standard étaient plus souvent renseignés que ceux nécessitant l'usage de techniques spéciales comme l'immunohistochimie (Tableau II). Cela s'explique en partie par l'indisponibilité de ces examens dans notre contexte. En effet, grâce à une étude menée entre 2011 et 2014, Nelson *et al.* ont montré que seulement 53% des pays d'Afrique subsaharienne disposaient de l'immunohistochimie et que les techniques de biologie moléculaire n’étaient disponibles que dans deux pays [21]. Dans notre étude, seules les femmes qui avaient les moyens d'assurer l'envoi de l’échantillon à l'extérieur (en France notamment) ont bénéficié d'une analyse immunohistochimique complète de leur prélèvement. Ce constat soulève la problématique de l'accès au diagnostic et au traitement du cancer du sein en Afrique subsaharienne. Dans les pays développés, les critères relatifs à l'analyse immunohistochimique sont renseignés dans plus de 90% des cas [4, 27].

#### Analyses de « niveau basique »

Seulement 2 CRA sur 3 contenaient les 4 éléments indispensables pour une analyse de base. L'atteinte ganglionnaire pN était moins souvent renseignée que tous les autres éléments rentrant en compte dans les analyses du niveau de base. Ce constat est identique à celui fait par Yesufe *et al.* en Éthiopie entre 2014 et 2016 [31]. Ce qui suggère une insuffisance de l'examen des ganglions pour une stadification correcte des malades. Cette situation pourrait s'expliquer par une insuffisance du nombre de ganglions retrouvés dans les curages axillaires. Dans notre série, 22% des curages axillaires dont le nombre de ganglions a été renseigné dans le CRA comportaient moins de 10 ganglions. La présence d'au moins 10 ganglions dans un curage axillaire est recommandée pour que le résultat soit recevable.

#### Analyses de «niveau moyen » et « niveau amélioré»

Les 3 éléments d'analyse dans un contexte de moyens limités étaient tous renseignés sur seulement 26% des CRA de type narratif et sur 86% des CRA de type synoptique. Les analyses de niveau amélioré étaient présentes sur la totalité des CRA de type synoptique. Bien que les CRA de type synoptique ne représentent que 7% de tous les CRA dans notre série, cette tendance suggère que l'usage d'un canevas pré-établi favorise un meilleur remplissage des CRA. Nos résultats sont similaires à ceux obtenus dans différents pays d'Afrique subsaharienne [7, 12, 31]. Dans les pays développés, l'usage des CRA de type synoptique a amélioré le renseignement des comptes rendus. En Australie, Austin *et al.* ont remarqué une nette augmentation de CRA conformes entre 1997 et 2004, après l'introduction des CRA synoptiques [4].

### Standardisation et comptes rendus synoptiques

Les résultats de notre étude et les données de la littérature montrent que la standardisation des FDA et CRA avec usage de check-lists permet d'augmenter significativement la probabilité de communiquer des informations plus complètes ou adéquates [12]. Idowu *et al*., dans l’étude la plus large que nous ayons trouvée, ont étudié 2125 CRA provenant de 86 établissements aux États-Unis. Les résultats montraient que 68,8% de tous les CRA comprenaient tous les éléments requis. Ce pourcentage était nettement plus élevé dans les structures qui faisaient usage de CRA synoptiques en comparaison des structures qui faisaient usage de CRA narratifs (88% contre 34%) [12]. Il serait donc judicieux que les sociétés savantes, de concert avec les autorités sanitaires des pays d'Afrique subsaharienne, éditent des recommandations et instaurent l'usage de CRA de type synoptique dans les laboratoires d'anatomie pathologique. Il faudrait en outre investir dans le renforcement des capacités humaines et matérielles de ces laboratoires.

## Conclusion

Un diagnostic histopathologique précis est l'un des piliers de la prise en charge du cancer du sein. Dans les pays à faibles ressources, des efforts doivent être faits pour s'assurer de la qualité des examens biologiques en général, et en particulier de la rédaction des comptes rendus d'examen anatomopathologique. Notre étude montre que les pratiques des professionnels de santé ne sont pas toujours conformes aux recommandations disponibles. L’édition de FDA et de CRA avec items prédéfinis, validés par les sociétés savantes devrait améliorer l'exhaustivité des renseignements fournis par ces documents. Une politique de santé publique et la recherche de financements publics doivent être mises en place pour permettre une meilleure accessibilité aux moyens diagnostiques et thérapeutiques, notamment l'immunohistochimie et les thérapies ciblées qui amélioreraient les pratiques des professionnels et par conséquent, la prise en charge des patientes et leurs chances de guérison.

## Considérations éthiques

Nous avons informé les différents chefs de service et les départements des affaires médicales des hôpitaux et laboratoires concernés par notre étude. Nous avons obtenu leurs autorisations et coopérations pour la collecte des données.

## Source de financement

Nous n'avons bénéficié d'aucun financement.

## Contribution des auteurs

Conception de l’étude: Freddy Houéhanou Rodrigue GNANGNON, Falilatou SEIDOU, Fèmi Perez ODIDI, Dansou Gaspard GBESSI. Collecte des données: Fèmi Perez ODIDI, Freddy Houéhanou Rodrigue GNANGNON, Falilatou SEIDOU, Christel Marie LALEYE, Arielle FLENON NAKOU.

Analyse des données: Fèmi Perez ODIDI, Christel Marie LALEYE, Freddy Houéhanou Rodrigue GNANGNON, Falilatou SEIDOU, Dismand Stephan HOUINATO.

Rédaction du manuscrit: Freddy Houéhanou Rodrigue GNANGNON, Christel Marie LALEYE, Falilatou SEIDOU, Fèmi Perez ODIDI. Supervision: Josiane Angéline TONATO BAGNAN, Justin Lewis DENAKPO, Dismand Stephan HOUINATO, Dansou Gaspard GBESSI.

## Liens d'intérêts

Les auteurs ne déclarent aucun lien d'intérêts.
